# Substrain-Dependent Differences in Doxorubicin-Induced Cardiotoxicity in Adult C57BL/6 Mice

**DOI:** 10.1007/s12012-025-10076-6

**Published:** 2025-12-23

**Authors:** Mary R. Daniel, Marianne K. O. Grant, Mohamed S. Dabour, Maria Razzoli, Fernando Souza-Neto, Jop H. van Berlo, Alessandro Bartolomucci, Beshay N. Zordoky

**Affiliations:** 1 Department of Experimental and Clinical Pharmacology, College of Pharmacy, University of Minnesota, Minneapolis, MN USA; 2 Department of Integrative Biology and Physiology, Medical School, University of Minnesota, Minneapolis, MN USA; 3 Cardiovascular Division, Department of Medicine, University of Minnesota, Minneapolis, MN USA; 4 Lillehei Heart Institute, University of Minnesota, Minneapolis, MN USA

**Keywords:** Doxorubicin, Cardiotoxicity, C57BL/6 Substrains, Transcriptomics

## Abstract

Doxorubicin (DOX)-induced cardiotoxicity is widely studied using C57BL/6 mice. However, potential substrain differences between C57BL/6J (6J) and C57BL/6N (6N) remain unexamined. Comparative studies are needed to improve preclinical reproducibility and translation. Fifteen-week-old male 6N and 6J mice received either DOX (8 mg/kg/week for 3 weeks) or saline. Body weight was monitored weekly, followed by echocardiography to assess cardiac function. Histopathology evaluated cardiac fibrosis, and bulk RNA sequencing identified differentially expressed genes, which were then validated by qPCR. DOX caused significant weight loss in 6N constituted by loss of both fat and fat-free mass, while 6J showed subtle changes. Echocardiography showed a significant ejection fraction decline only in 6J, while cardiac output, stroke volume, and heart weight were reduced in both. Gene expression analysis revealed significant upregulation of atrial natriuretic peptide and myosin heavy chains in both substrains, with B-type natriuretic peptide upregulation in 6N only. Transcriptomic analysis identified 518 upregulated and 203 downregulated genes in 6N, and 406 upregulated and 445 downregulated genes in 6J, with 113 and 115 commonly upregulated and downregulated genes, respectively. Gene set enrichment analysis revealed that DOX-treated 6N mice showed enrichment of TGF-beta signaling pathways, and suppression of IL-6–JAK–STAT3, epithelial-mesenchymal transition, and myogenesis pathways. In contrast, 6J exhibited enrichment of oxidative phosphorylation, p53 pathway, and reactive oxygen species pathways. Both substrains showed enrichment of apoptosis, unfolded protein response, TNFα signaling via NF-κB, and heme metabolism pathways and downregulation of Hedgehog Signaling and Interferon Gamma Response pathways. This study demonstrates substrain-dependent differences in DOX-induced cardiotoxicity, highlighting how genetic background affects cardiac function, gene expression, and pathway enrichment.

**Graphical Abstract:**

Substrain-specific differences in doxorubicin-induced cardiotoxicity

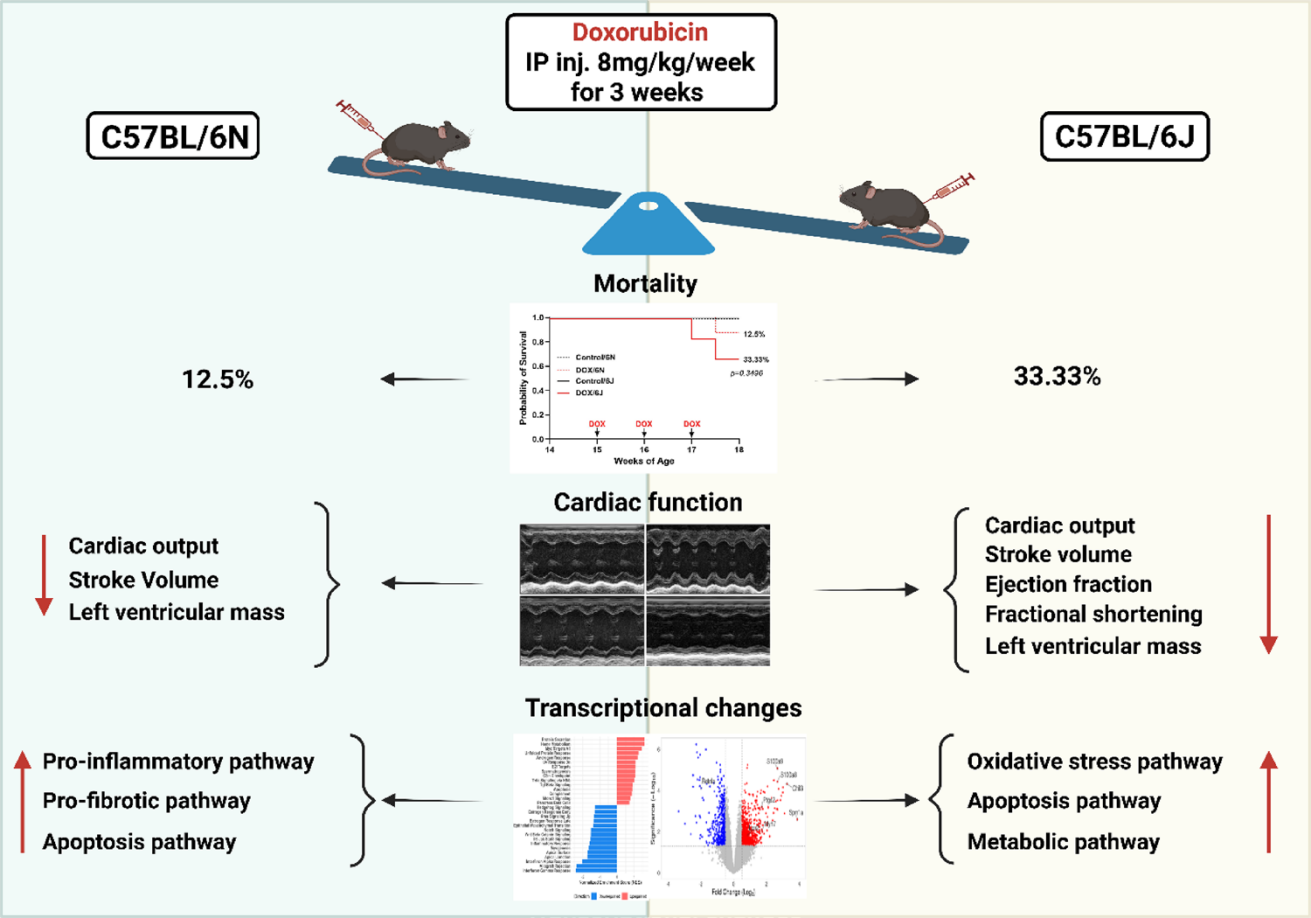

**Supplementary Information:**

The online version contains supplementary material available at 10.1007/s12012-025-10076-6.

## Introduction

Anthracycline-induced cardiotoxicity, particularly from doxorubicin (DOX), remains a significant challenge in cancer therapy, as its use is limited by the risk of irreversible heart damage [1]. DOX-induced cardiotoxicity (DIC) serves as a widely accepted preclinical model to study mechanisms underlying chemotherapy-associated cardiovascular complications and explore potential therapeutic interventions [2, 3]. Rodent models, particularly mice, are indispensable tools in cardio-oncology research due to their genetic tractability and reproducibility [4]. However, emerging evidence underscores the influence of genetic background, including mouse substrain variability, on phenotypic responses such as cardiovascular outcomes [5].

The C57BL/6 mouse strain is among the most extensively utilized in biomedical research, making it a preferred choice for testing drug-like compounds, and generating transgenic models of human diseases [6]. However, genetic and phenotypic differences between its substrains, C57BL/6N (6N) and C57BL/6J (6J), contribute to variability in disease susceptibility [6–9]. Notably, 6J carries a spontaneous deletion in the *Nnt* gene, impairing mitochondrial NADPH production and redox balance [10, 11], while 6N harbors a null mutation in *Mylk3*, linked to age-related dilated cardiomyopathy, reduced cardiac contractility [12], and also carries the *Crb1 rd8* mutation responsible for retinal degeneration [13]. Functional studies indicate that these genetic differences contribute to distinct cardiovascular phenotypes. In transverse aortic constriction-induced pressure overload, 6N mice show greater maladaptive remodeling and functional decline than 6J mice [14, 15]. Likewise, in angiotensin II-induced remodeling, 6N mice exhibit increased fibrosis and higher baseline AT2R expression [14, 16]. Together, these findings suggest that substrain-specific mutations influence oxidative stress, extracellular matrix regulation, and cardiac stress responses, potentially associated with differential susceptibility to DIC [15, 17, 18].

Nevertheless, no study to date has directly compared the susceptibility of 6N and 6J substrains to DOX induced cardiotoxicity. This critical gap in knowledge limits our understanding of genetic and phenotypic variability in response to anthracycline treatment, which could influence translationally-relevant preclinical outcomes. In this study, we systematically evaluated substrain-dependent vulnerability to DIC by assessing cardiac function, histological changes, and molecular markers of cardiac injury to provide novel insights into the impact of genetic background on anthracycline-induced cardiac responses.

## Materials and Methods

### Animal Care and Housing

Twelve-week-old male C57BL/6N (6N) and C57BL/6J (6J) mice were obtained from Charles River Laboratories (Wilmington, MA, USA) and Jackson Laboratories (Bar Harbor, ME, USA), respectively. Upon arrival, mice were acclimated prior to the initiation of experimental procedures. Animals were housed under standard conditions (22 ± 2 °C, 12:12 h light-dark cycle) with ad libitum access to a standard chow diet (2918 Teklad) and water. All animal protocols were approved by the Institutional Animal Care and Use Committee (IACUC) at the University of Minnesota (Protocol ID: 2106–39176 A).

### Study Design and Experimental Groups

**Fig. 1:**
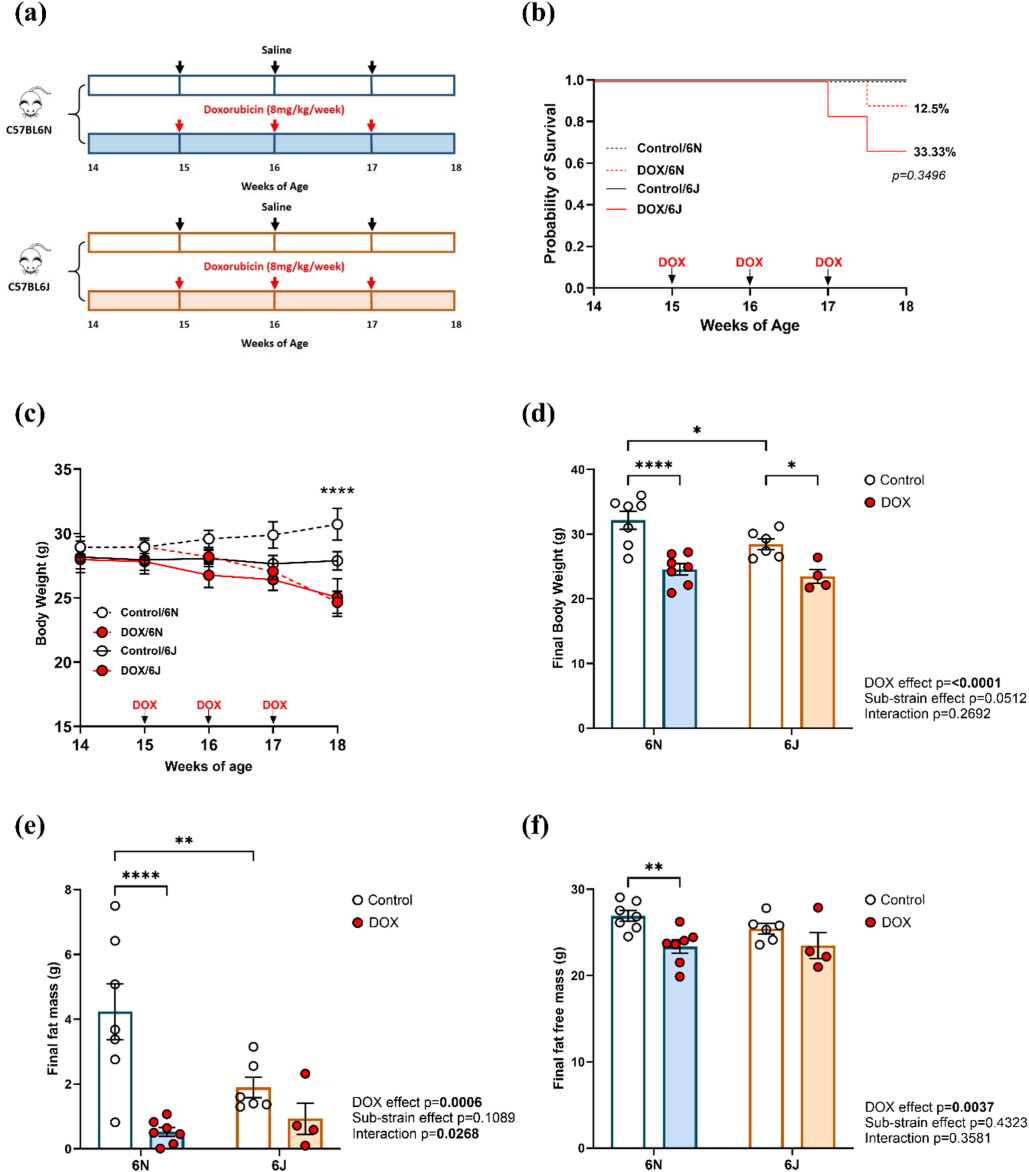
Substrain-specific effects of DOX on survival, body weight, and composition in C57BL/6 mice. **a** Graphical representation of the experimental design, **b** Kaplan-Meier survival analysis (12.5% and 33.33% indicates mortality in DOX/6N and DOX/6J groups respectively), **c** Body weights across weeks of age (*indicates a significant difference between Control/6N and DOX/6N groups by ordinary one-way ANOVA with mixed-effects analysis), **d** Final body weight, **e** Final fat mass, and **f** Final fat free mass. Values are represented as means ± SEMs (*n* = 4–7 per group). Statistical analysis was performed by two-way ANOVA with Tukey’s post-hoc analysis as appropriate (*p < 0.05, **p < 0.01, ****p < 0.0001). Detailed statistical analyses are provided in Supplementary Table 2

Male mice from each substrain (6N and 6J) were randomly assigned to one of two treatment groups: DOX or saline. The experimental design is illustrated in Fig. 1a and comprised the following groups: (1) Control/6N–6N mice treated with saline (*n* = 7); (2) DOX/6N–6N mice treated with DOX (*n* = 8); (3) Control/6J–6J mice treated with saline (*n* = 6); and (4) DOX/6J–6J mice treated with DOX (*n* = 6). In this study, 15-week-old C57BL/6 mice were used, representing a physiologically mature yet non-aged stage corresponding to young adulthood in humans (~ 25–30 years) [19, 20]. This age reflects the clinical population most often treated with doxorubicin, such as adults with leukemias, lymphomas, and soft-tissue sarcomas [21–23]. At 15 weeks of age, mice received intraperitoneal injections of DOX at a dose of 8 mg/kg once weekly for three consecutive weeks (cumulative 24 mg/kg). This regimen corresponds to a human-equivalent dose of approximately 72 mg/m², calculated using the FDA-recommended body surface area (BSA) allometric scaling method (Km: mouse = 3; human = 37) [24] and falls within the clinically relevant range for adult patients [25, 26]. The selected dose is consistent with previously validated murine models of DIC that employ 5–10 mg/kg weekly for 3–4 weeks, producing reproducible cardiac dysfunction and remodeling phenotypes [2, 27, 28]. Control mice received an equivalent volume of sterile saline. Body weight was recorded weekly to monitor general health and systemic toxicity. Six days after the last dose of DOX or saline injection, cardiac function was assessed by transthoracic echocardiography. The following day, mice were humanely euthanized under isoflurane anesthesia followed by decapitation. Hearts were immediately excised, rinsed in cold phosphate-buffered saline, flash-frozen in liquid nitrogen, and stored at − 80 °C for subsequent analyses.

### Body Composition Analysis

Body composition was assessed four days following the final administration of DOX or saline using the EchoMRI™ 3-in-1 quantitative magnetic resonance system (EchoMRI LLC, Houston, TX, USA). Measurements included total fat mass and fat-free mass.

### Echocardiographic Assessment of Cardiac Function

Cardiac function was evaluated via transthoracic echocardiography six days following the final administration of DOX or saline. Echocardiography was performed using the Vevo 2100 high-resolution imaging system (VisualSonics Inc., Toronto, ON, Canada) equipped with an MS400 transducer. Mice were anesthetized with 3% isoflurane for induction and maintained at 1–2% isoflurane throughout the procedure. Animals were positioned supine on a heated physiological monitoring platform to maintain normothermia and enable real-time monitoring of heart rate (HR) and respiratory rate. Parasternal short-axis images of the left ventricle were acquired in M-mode at the level of the papillary muscles. Endocardial and epicardial borders were manually traced across 3–4 consecutive cardiac cycles. Cardiac parameters including cardiac output (CO), ejection fraction (EF), fractional shortening (FS), stroke volume (SV), and left ventricular (LV) mass were calculated using the dedicated cardiac analysis software integrated within the Vevo 2100 system.

### Histopathological Analysis

Left ventricle (LV) heart specimens were obtained, fixed in 10% neutral buffered formalin, and subsequently embedded in paraffin. Sections, measuring four microns in thickness, were subjected to Masson’s trichrome staining to gauge the extent of fibrosis. Collagen deposition was quantified based on the area stained in blue, which was measured relative to the total tissue area identified by the red staining. Quantification was performed using CellProfiler software. To evaluate cardiomyocyte surface area (CSA), heart sections were deparaffinized and incubated with Wheat Germ Agglutinin (WGA) diluted 1:200 in PBS for 1 h, followed by PBS washes and mounting. Fluorescence images were captured using an upright AXIO M2 microscope (Zeiss).

### RNA Extraction

Total RNA was isolated from approximately 20 mg of frozen ventricular tissue using 300 µL of TRIzol™ reagent (Invitrogen, Thermo Fisher Scientific, Waltham, MA, USA) following the manufacturer’s protocol. Homogenization was carried out using a Biogen Pro200 homogenizer (Pro Scientific Oxford, CT, USA) to ensure complete lysis of tissue. RNA concentration and purity were assessed by measuring absorbance at 260 nm using a NanoDrop Lite Plus spectrophotometer (Thermo Fisher Scientific, Waltham, MA, USA). Samples were stored at − 80 °C until further processing.

### Bulk RNA-Seq and Bioinformatics Analysis

#### Sample Preparation and Quality Assessment

Total RNA extracted from ventricular tissue was further purified using the RNeasy Plus Micro Kit (Qiagen, Germantown, MD), which includes a genomic DNA elimination step to ensure removal of contaminating host DNA. All procedures were performed in accordance with the manufacturer’s instructions. Purified RNA samples were quantified using a RiboGreen fluorescence-based assay (Thermo Fisher Scientific), and RNA integrity was assessed by capillary electrophoresis using the Agilent 2100 BioAnalyzer system (Agilent Technologies, Santa Clara, CA, USA), which generated RNA Integrity Numbers (RIN). Samples were required to meet minimum quality control criteria, including a yield of > 500 ng and a RIN ≥ 8, to proceed to downstream applications. RNA samples passing QC were subsequently used for Illumina library preparation and sequencing.

##### Library Creation

Total RNA samples that passed quality control were used for library preparation using either the TruSeq RNA Sample Preparation Kit (Cat. #RS-122–2001 or RS-122–2002) or the TruSeq Stranded mRNA Library Prep Kit (Cat. #RS-122–2101; Illumina, San Diego, CA, USA), following the manufacturer’s protocol. Briefly, polyadenylated mRNA was isolated from a standardized input of total RNA using oligo-dT magnetic beads, fragmented, and reverse transcribed to generate double-stranded cDNA. The cDNA was then end-repaired, A-tailed, and ligated to indexed, molecularly barcoded adapters. Libraries were amplified by 15 cycles of PCR. The final library size distribution was assessed using capillary electrophoresis (e.g., Agilent BioAnalyzer), and concentrations were determined using fluorometric quantification (PicoGreen) and quantitative PCR (qPCR). Indexed libraries were normalized, pooled, and size-selected to approximately 320 bp using the PippinHT system (Sage Science, Beverly, MA, USA) for downstream high-throughput sequencing.

##### Cluster Generation and Sequencing

Pooled libraries were denatured and diluted to the appropriate concentration for clustering and loaded onto a NovaSeq paired-end flow cell (Illumina, San Diego, CA, USA). Cluster generation was performed on-board the instrument, followed by sequencing using Illumina’s two-color sequencing-by-synthesis (SBS) chemistry. After completion of the first read (Read 1), a 7-base index read was performed for single-indexed libraries. For dual-indexed libraries, two separate index reads of 8 or 10 bases were performed. Subsequently, the complementary strand of the library fragments was synthesized to enable generation of the second read (Read 2), completing paired-end sequencing.

##### Primary Analysis and De-multiplexing

Base call (.bcl) files were generated in real-time during sequencing using Illumina’s Real-Time Analysis (RTA) software. These files, along with associated run folders, were streamed to secure servers maintained by the Minnesota Supercomputing Institute (MSI), University of Minnesota. Primary data processing, including base calling and demultiplexing, was performed using Illumina’s bcl-convert software (version 4.0.3). This process resulted in the generation of demultiplexed FASTQ files, which were subsequently made available to client accounts for downstream analysis using mapping and alignment tools of their choice.

#### RNA-Seq Data Processing, Differential Expression, and Pathway Analysis

Quality control, alignment, and gene quantification were performed using the CHURP pipeline at the Minnesota Supercomputing Institute (MSI), University of Minnesota. Paired-end 2 × 150 bp FASTQ files from 16 samples (average of ~ 37.5 million reads per sample) were initially processed using Trimmomatic (v0.33), employing a 3 bp sliding window from the 3’ end with a minimum quality threshold of Q30 (“-q” option enabled). FastQC was used to assess the quality of the raw sequencing reads for each sample. Trimmed reads were aligned to the mouse reference genome (GRCm39) using HISAT2 (v2.1.0). Gene-level quantification was performed with FeatureCounts, producing raw read counts for each gene. Differential expression analysis was conducted using the edgeR package (negative binomial model in R), implemented within CHURP. Volcano plots were generated using the VolcaNoseR platform, applying log₂ fold change cutoff of > 0.5 and *p* < 0.05.

To investigate biological pathway perturbations, Gene Set Enrichment Analysis (GSEA) was conducted on the ranked gene list, ordered by log₂ fold change values. Only genes with a log₂ fold change of > 0.5 and an associated p-value < 0.05 were retained for the analysis. Gene identifiers were matched to curated Hallmark gene sets from the Molecular Signatures Database (MSigDB) using the msigdbr R package. Enrichment analysis was performed using the fgsea package with 1,000 permutations to assess statistical significance. Only gene sets containing 10 to 300 genes were included. Normalized Enrichment Scores (NES) were calculated to account for gene set size, and FDR-adjusted q-values were computed using the Benjamini-Hochberg method. The top 15 upregulated and top 15 downregulated Hallmark pathways were visualized using ggplot2, with red and blue bars representing upregulated and downregulated pathways, respectively. For visualization of gene expression patterns, a heatmap of the top 50 significantly differentially expressed genes was generated based on DESeq2 analysis. Genes were selected based on an adjusted p-value < 0.05 and a log₂ fold change > 0.5. Expression values were log₂-transformed and Z-score normalized across genes. Hierarchical clustering using Euclidean distance and complete linkage was applied to identify patterns of gene expression similarity. Experimental groups were annotated with DOX-treated samples displayed in coral red and saline controls in teal blue. The heatmap was created using the pheatmap R package.

#### cDNA Synthesis and Quantitative Real-Time PCR

First-strand cDNA was synthesized from 1.5 µg of total RNA using the High-Capacity cDNA Reverse Transcription Kit (Applied Biosystems, Thermo Fisher Scientific) following the manufacturer’s protocol. Quantitative real-time PCR (qRT-PCR) was performed using SYBR Green chemistry on an Applied Biosystems QuantStudio 5 Real-Time PCR System (Thermo Fisher Scientific) with 384-well optical reaction plates. The thermal cycling conditions were as follows: initial denaturation at 95 °C for 10 min, followed by 40 cycles of denaturation at 95 °C for 15 s and annealing/extension at 60 °C for 1 min. Gene-specific primers (Supplementary Table 1) were used to assess mRNA expression. Relative gene expression was calculated using the comparative ΔΔCT method, with *Actb* serving as the internal control. Expression values were normalized to the saline-treated control group within each respective substrain. Primer specificity and the absence of non-specific amplification were confirmed through melting curve analysis.

### Statistical Analysis

All data were analyzed using GraphPad Prism software (version 10.1.2; GraphPad Software, La Jolla, CA, USA) and are expressed as mean ± standard error of the mean (SEM). Statistical significance was assessed using one-way or two-way analysis of variance (ANOVA) for repeated measures, followed by Tukey’s Honestly Significant Difference (HSD) post hoc test, or two-way mixed-effects ANOVA with Tukey’s post hoc correction, as appropriate. A p-value of < 0.05 was considered statistically significant. The specific statistical tests applied to each dataset are detailed in the corresponding Fig. legends.

## Results

### Substrain-Specific Effects of DOX on Survival, Body Weight, and Composition in 6N and 6J Mice

The experimental timeline is depicted in Fig. 1a, where mice were treated with saline or DOX (8 mg/kg/week) for three weeks starting at 15 weeks of age. No mortality was observed in saline-treated groups of either substrain, while DOX treatment resulted in 12.5% mortality in 6N mice (1 out of 8) and 33.3% mortality in 6J mice (2 out of 6), suggesting a trend toward higher susceptibility to DOX toxicity in 6J mice. However, this difference was not statistically significant (*p* = 0.3496) (Fig. 1b). Body weight trajectories and final body weights were significantly higher in 6N mice compared to 6J mice within the control groups (Fig. 1c and d), potentially due to a greater fat mass observed in Control/6N mice (Fig. 1e). Final body weights were significantly decreased in both substrains following DOX treatment (Fig. 1d). In 6N mice, this reduction in body weight was associated with significant decreases in both fat mass (Fig. 1e) and fat-free mass (Fig. 1f). In contrast, DOX treatment did not significantly alter body composition in 6J mice (Fig. 1e and f). These results highlight substrain-specific differences in the systemic toxic effects of DOX.

### Substrain Specific Effects of DOX on Cardiac Function in C57BL/6 Mice

**Fig. 2:**
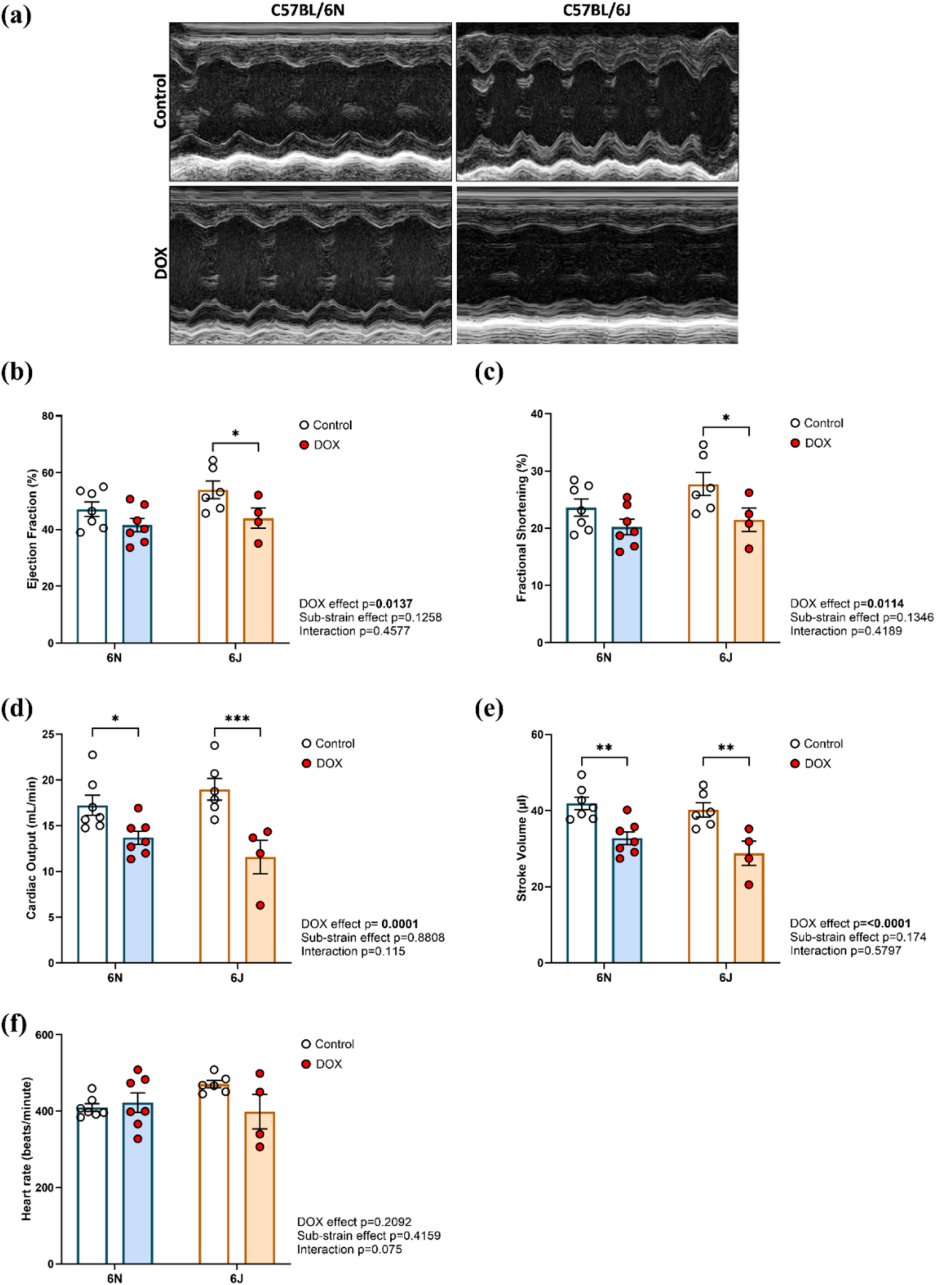
Substrain-specific effects of DOX on cardiac function in C57BL/6 mice. **a** Representative M-Mode images of parasternal short axis view of the heart, **b** Ejection fraction, **c** Fractional shortening, **d** Cardiac output, **e** Stroke volume, and **f** Heart rate. Values are represented as means ± SEMs (*n* = 4–7 per group). Statistical analysis was performed by two-way ANOVA with Tukey’s post-hoc analysis as appropriate (*p < 0.05, **p < 0.01, ***p < 0.001). Detailed statistical analyses are provided in Supplementary Table 2

Echocardiography was performed 6 days following the last dose of DOX. Representative M-mode images obtained from individual mice in each of the groups are shown in Fig. 2a. Compared to their respective controls, DOX significantly reduced EF (Fig. 2b) and FS (Fig. 2c) in the 6J, but not in the 6N substrain. CO (Fig. 2d) and SV (Fig. 2e) were significantly reduced by DOX in both the substrains. No significant differences in HR were observed between any of the groups (Fig. 2f).

### Substrain-Specific Effects of DOX on Cardiac Morphometry in C57BL/6 Mice

**Fig. 3:**
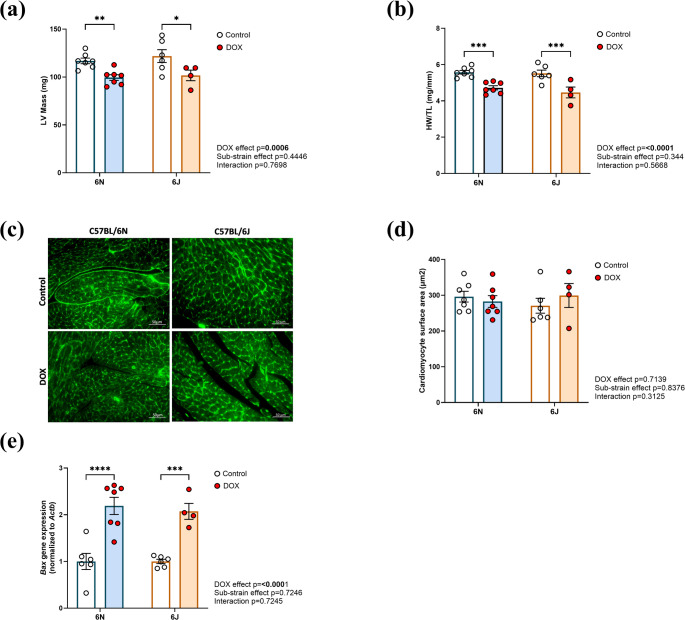
Substrain-specific effects of DOX on cardiac morphometry in C57BL/6 mice. **a** LV mass, **b** Heart weight/tibial length (HW/TL), **c **Representative wheat germ agglutinin (WGA) stained images of ventricular heart sections highlighting cardiomyocyte membrane boundaries. Scale bar = 50 μm. **d** Quantification of cardiomyocyte surface area (CSA, µm²), **e** The mRNA expression of Bcl-2-associated X protein (*Bax*), normalized to *Actb* (*β-actin*). Values are represented as means ± SEMs (*n* = 4–7 per group). Statistical analysis was performed by two-way ANOVA with Tukey’s post-hoc analysis as appropriate (*p < 0.05, **p < 0.01, ***p < 0.001, ****p < 0.0001). Detailed statistical analyses are provided in Supplementary Table 2

DOX treatment resulted in LV atrophy, as evidenced by reductions in LV mass (Fig. 3a) and heart weight to tibia length ratio (HW/TL) (Fig. 3b) in both the substrains. All other parameters of cardiac morphometry, including left ventricular anterior wall thickness at systole (LVAW; s) and diastole (LVAW; d), left ventricular posterior wall thickness at systole (LVPW; s) and diastole (LVPW; d), left ventricular internal diameter at systole (LVID; s) and diastole (LVID; d), and left ventricular volume at systole (LV Vol; s) and diastole (LV Vol; d), are summarized in Supplementary 1 Table 2. No significant differences were observed in any of these parameters. To further examine these structural changes at the cellular level, WGA staining was performed to measure CSA (Fig. 3c, d). No significant differences in CSA were observed between substrains following DOX treatment, suggesting that the observed cardiac atrophy may not be due to a reduction in individual cardiomyocyte size. Further, gene expression analysis showed a significant increase in the pro-apoptotic Bcl-2–associated X protein (*Bax*) gene in both substrains (Fig. 3e), indicating activation of apoptotic pathways are likely contributing to the observed cardiac atrophy.

### Substrain-Specific Effects of DOX on Markers of Cardiac Stress and Remodeling in C57BL/6 Mice

**Fig. 4:**
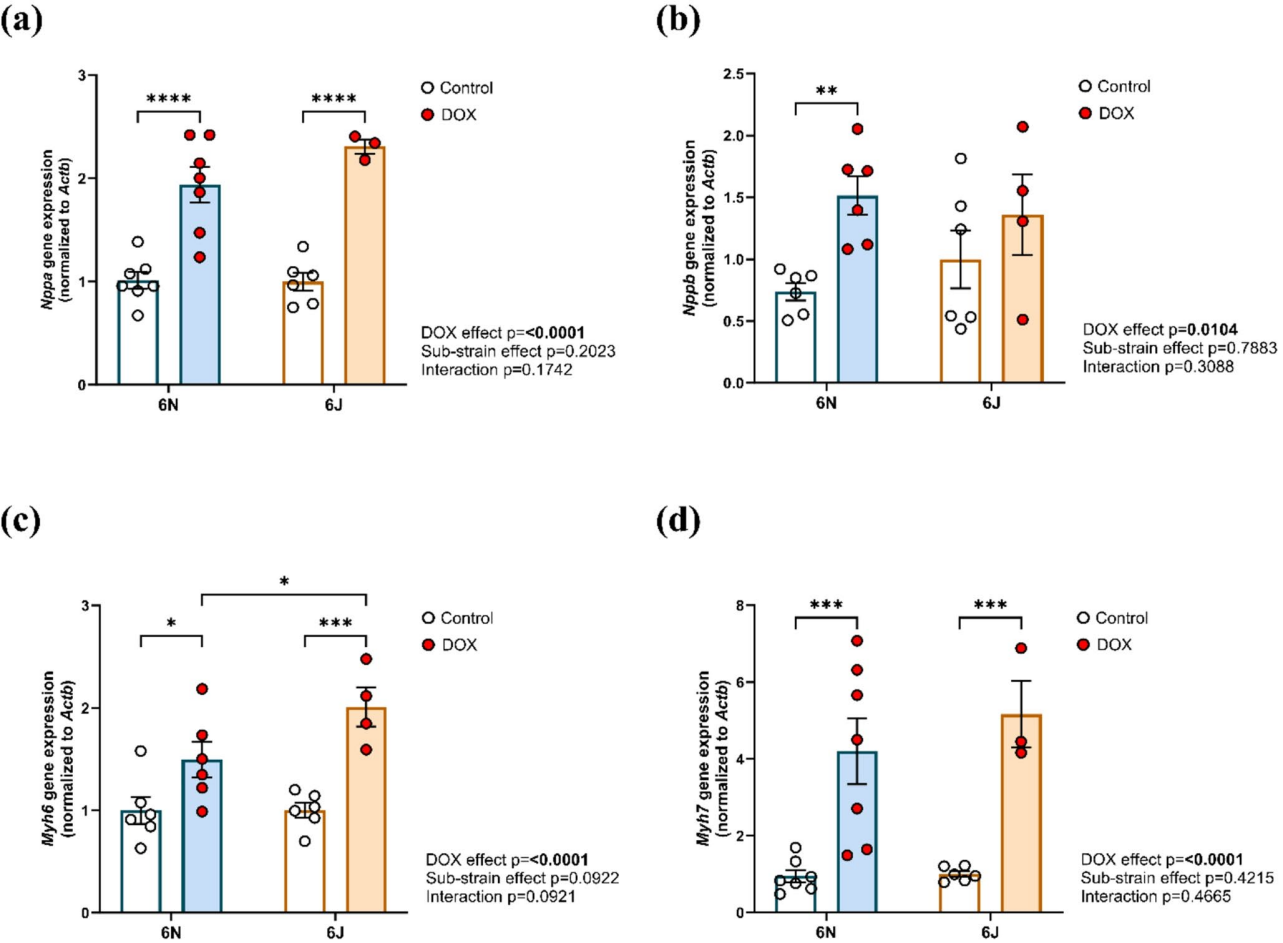
Substrain-specific effects of DOX on markers of cardiac stress and remodeling in C57BL/6 mice. The mRNA expression of **a** Natriuretic peptide A (*Nppa*), **b** Natriuretic peptide B (*Nppb*), **c** Myosin heavy chain 6 (*Myh6*), and **d** Myosin heavy chain 7 (*Myh7*), normalized to *Actb* (*β-actin*). Values are represented as means ± SEMs (*n* = 4–7 per group). Statistical significance of pairwise comparisons was determined by two-way ANOVA with Tukey’s post-hoc analysis as appropriate (*p < 0.05, **p < 0.01, ***p < 0.001, ****p < 0.0001). Detailed statistical analyses are provided in Supplementary Table 3

Markers for cardiac stress were assessed by qPCR to determine if the observed changes in cardiac function after DOX treatment are associated with pathological changes in gene expression in the heart (Fig. 4a–d). A significant increase in *Nppa* gene expression was observed in both substrains of mice following DOX treatment (Fig. 4a). Another critical marker of cardiac stress *Nppb* was significantly increased in the 6N substrain, while in 6J it increased with DOX but not significantly (Fig. 4b). Expression levels of *Myh6* (Fig. 4c) and *Myh7* (Fig. 4d), which encode for cardiac α-myosin heavy chain (α-MHC) and β-myosin heavy chain (β-MHC), respectively, were significantly elevated in DOX-treated groups in both substrains, suggesting increased maladaptive remodeling associated with DIC.

### Substrain-Specific Transcriptional Changes Induced by DOX

**Fig. 5:**
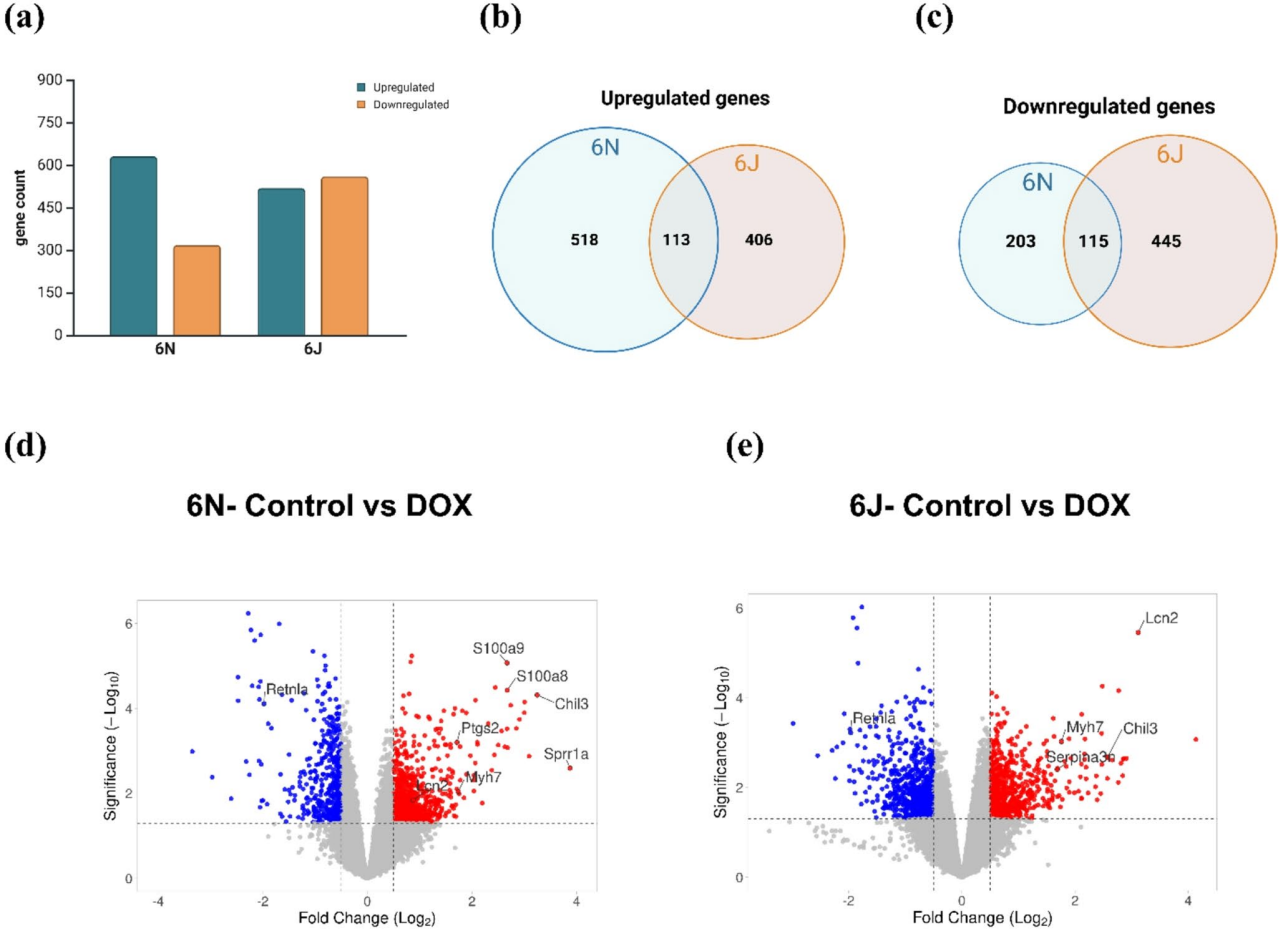
Substrain-specific transcriptional changes induced by DOX. Bulk RNA-seq analysis of 6N and 6J substrains in response to DOX treatment. **a** The number of up- and down regulated genes in response to DOX treatment in 6N and 6J substrains. **b** & **c** Venn diagrams showing the distribution of up- and down regulated genes in 6N and 6J substrains. Volcano plot of the DEGs using log₂ fold change cutoff of > 0.5 and *p* < 0.05 in (**d**) Control vs. DOX in 6N substrain and **e** Control vs. DOX in 6J substrain. Significantly upregulated genes are shown in red, while significantly downregulated genes are shown in blue

To determine the transcriptional changes associated with DOX treatment in C57BL/6 substrains, bulk RNA sequencing was conducted on heart tissue samples from mice (*n* = 4 per group). We analyzed differential gene expression across two experimental comparisons: Control vs. DOX in 6N and 6J substrains respectively. Differential gene expression analysis revealed a greater number of changes in the 6N substrain compared to the 6J substrain in the heart. Specifically, 518 genes were upregulated in 6N, while 406 genes were upregulated in 6J. In contrast, 6N exhibited fewer downregulated genes (203 genes) compared to 6J, which had 445 downregulated genes. Additionally, a subset of differentially expressed genes (DEGs) overlapped between the two substrains. Venn diagram identified 113 genes that were commonly upregulated, while 115 genes were commonly downregulated following DOX treatment (Fig. 5a–c) (Supplementary 2). These findings indicate that while both substrains undergo significant transcriptional alterations in response to DOX, the extent and direction of these changes differ, potentially influencing substrain-specific susceptibility to DIC. Volcano plots (Fig. 5d and e) illustrate significantly upregulated and downregulated genes in each substrain. A subset of cardiovascular-relevant genes is annotated in the volcano plots, represent distinct functional categories. These include genes involved in inflammation and cellular injury (*S100a8/a9*,* Ptgs2*), cardiac stress and remodeling (*Sprr1a*,* Myh7*), cellular stress responses (*Serpina3n*,* Lcn2*), immune modulation and remodeling (*Chil3*), and tissue repair (*Retnla*).

**Fig. 6:**
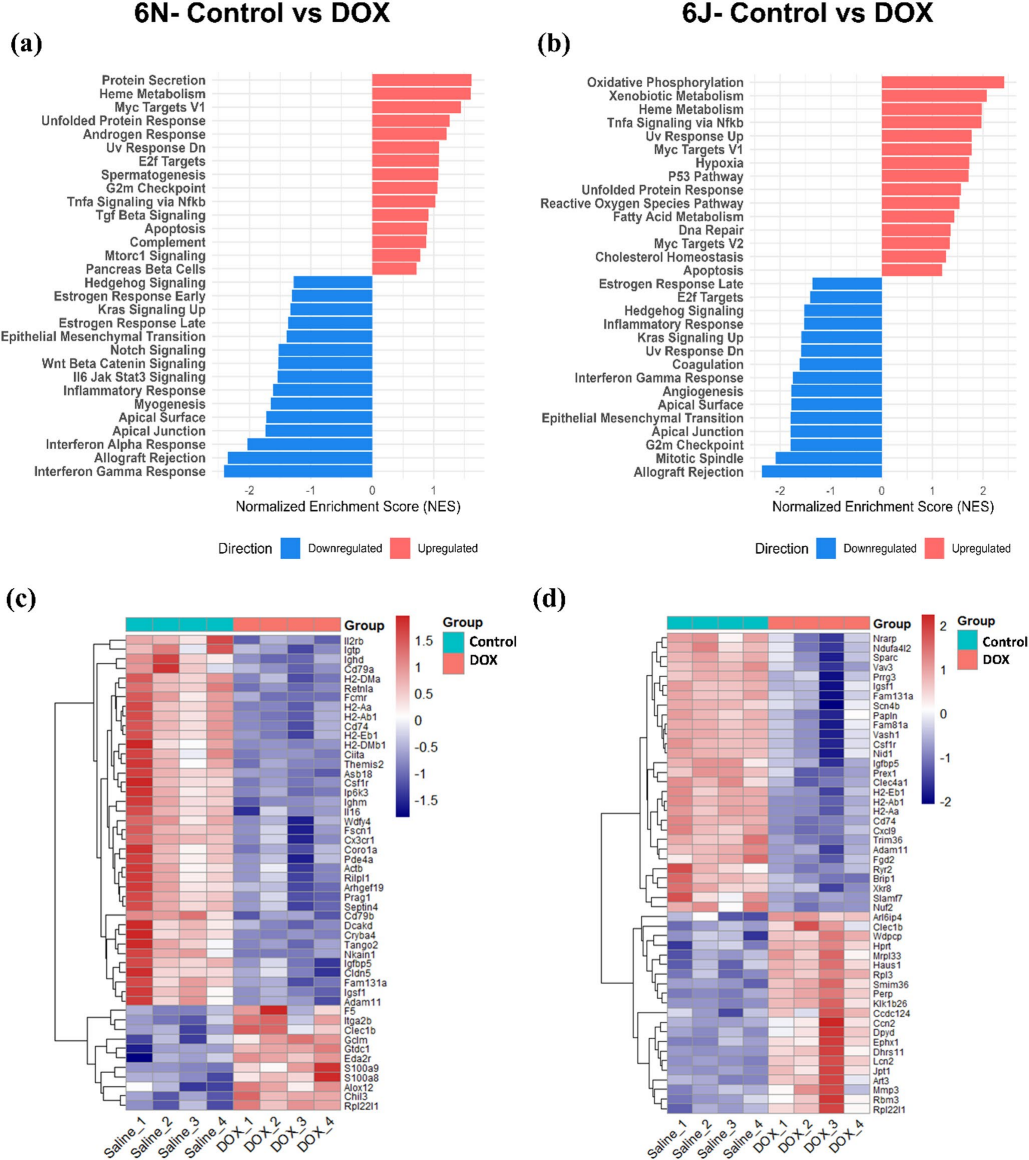
Substrain-specific transcriptional changes induced by DOX. Bulk RNA-seq analysis of 6N and 6J substrains in response to DOX treatment. GSEA plot comparing **a** 6N-Control vs. DOX, and **b** 6J-Control vs. DOX. The plot displays normalized enrichment scores (NES) for hallmark gene sets, categorized by their direction of regulation. Upregulated pathways in the DOX group are shown in red, while downregulated pathways are shown in blue. The NES represents the degree of enrichment for each pathway, with positive scores indicating upregulation and negative scores indicating downregulation in the DOX-treated group. Clustering of differentially expressed genes. Heatmaps demonstrating clustering of the top 50 differentially expressed in the heart of DOX treated animals as compared to control (heatmaps FC ≥ 0.5, *P* < 0.05) in **c** 6N and **d** 6J substrains

To further elucidate the biological pathways impacted by DOX treatment, we performed GSEA to identify significantly activated and suppressed pathways in each substrain (Fig. 6a and b). Among the commonly upregulated pathways in both 6N and 6J mice were heme metabolism, MYC targets V1, unfolded protein response, TNFα signaling via NF-κB, and apoptosis, indicating activation of stress, inflammatory, and cell death-related mechanisms. Downregulated pathways shared between the two substrains included Hedgehog signaling, Estrogen response late, kras signaling up, epithelial-mesenchymal transition (EMT), inflammatory response, apical surface, apical junction, allograft rejection, and interferon gamma response, suggesting suppression of developmental, epithelial integrity, and immune-related processes. Strain-specific responses were also observed. In 6N mice, pathways distinctly upregulated by DOX included protein secretion, androgen response, UV response DN, E2F targets, spermatogenesis, G2M checkpoint, complement, mTORC1 signaling, and pancreatic beta cell signaling. Pathways downregulated specifically in 6N included estrogen response early, KRAS signaling up, Notch signaling, Wnt/β-catenin signaling, IL-6/JAK/STAT3 signaling, myogenesis, and interferon alpha response. In contrast, 6J mice showed distinct upregulation of pathways such as oxidative phosphorylation, xenobiotic metabolism, UV response UP, hypoxia, p53 pathway, reactive oxygen species pathway, fatty acid metabolism, DNA repair, MYC targets V2, and cholesterol homeostasis, while E2F targets, UV response dn, coagulation, angiogenesis, G2M checkpoint, and mitotic spindle were among the pathways specifically downregulated. Overall, 6N mice exhibit a dysregulated immune and proliferative response, with signs of cell cycle stress, inflammation, and impaired tissue repair mechanisms. While both substrains showed upregulation of apoptotic pathways, 6J mice demonstrated a stronger mitochondrial and DNA damage response, accompanied by enhanced oxidative stress. These findings underscore substrain-specific differences in the molecular response to DOX treatment.

Heatmap analyses of the top 50 DEGs, arranged based on adjusted p-value, showed that in 6N mice (Fig. 6c) DOX led to upregulation of *S100a8*,* S100a9*,* Chil3*, *Alox12*,* Ccl5*, and *Adam11*-genes associated with inflammatory responses, innate immunity, oxidative stress, and metabolic alterations. Conversely, significantly downregulated genes included *Retnla*,* Il2rb*,* Igtp*,* Fcgr*,* Ighd*,* Ighm*,* Cd79a*, and *Cd79b* associated with tissue repair, immune regulation, particularly B cell function and interferon signaling. In 6J mice (Fig. 6d), upregulated genes such as *Clec1b*,* Lcn2*, *Mmp3*,* Ccn2*,* Dpyd*,* Ephx1*,* Dhrs11*,* Perp*, and *Art3* are associated with immune activation, oxidative stress response, extracellular matrix remodeling, and apoptosis. Genes like *Rpl3*, *Mrpl33*, *Haus1*, *Smim36*, and *Rbm3* relate to ribosomal function and cellular stress adaptation. Downregulated genes such as *Nrarp*,* Ndufa4l2*,* Sparc*,* Vav3*,* Csf1r*,* Nid1*,* Igfbp5*,* Ryr2*, and *Vash1* are linked to mitochondrial function, immune signaling, vascular regulation, and calcium handling. Additionally, decreased expression of MHC class II genes (*H2-Eb1*,* H2-Ab1*,* H2-Aa*), *Cd74*, and *Cxcl9* indicates suppressed antigen presentation and adaptive immune responses.

### Validating Differentially Expressed Genes by Quantitative Real Time-PCR

**Fig. 7:**
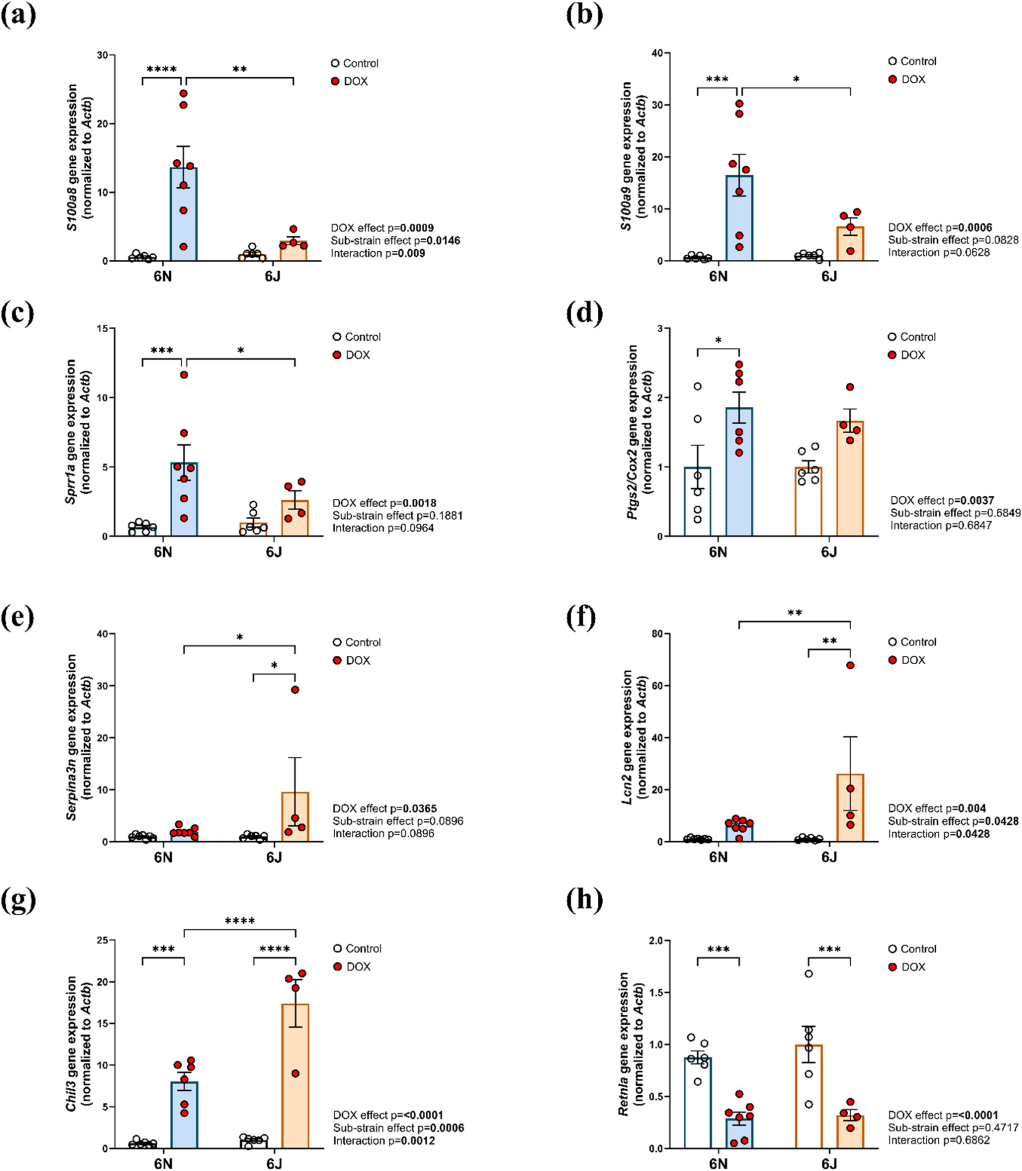
Validating DEGs by quantitative real time-PCR. The mRNA expression of **a** S100 calcium-binding protein A8 (*S100a8*), **b** S100 calcium-binding protein A9 (*S100a9*), **c** Small proline-rich protein 1 A (*Sprr1a*), **d** Prostaglandin-Endoperoxide Synthase 2/Cyclooxygenase-2 (*Ptgs2/Cox-2*), **e** Serine protease inhibitor A3N (*Serpina3n*), **f** Lipocalin-2 (*Lcn2*), **g **Chitinase-like 3 (*Chil3*), and **h** Resistin-like alpha (*Retnla*) in the hearts of 6N and 6J substrains following DOX treatment. Values are represented as means ± SEMs. *n* = 4–7 per group. Statistical significance of pairwise comparisons was determined by two-way ANOVA with Tukey’s post-hoc analysis as appropriate (*p < 0.05, **p < 0.01, ***p < 0.001, ****p < 0.0001). Detailed statistical analyses are provided in Supplementary Table 3

To validate the differential gene expression findings and further explore the distinct molecular adaptations to DOX, we performed quantitative real-time PCR on selected genes implicated in cardiac stress, inflammation, and injury. Although *S100a8* and *S100a9* can exhibit both pro- inflammatory [29] and anti-inflammatory [30] roles depending on the specific conditions and disease settings, in the setting of DIC, they are known to act predominantly as pro-inflammatory mediators [31]. In 6N mice, S*100a8* and *S100a9*, genes known to promote inflammation, were significantly upregulated following DOX treatment, while their expression remained markedly lower in 6J mice (Fig. 7a, b). Similarly, *Sprr1a*, a gene linked to cardiac stress and remodeling, was significantly upregulated in 6N but not in 6J (Fig. 7c), further supporting the pro-apoptotic and maladaptive remodeling signature. Furthermore, *Ptgs2/COX-2* (Fig. 7d) was significantly upregulated in 6N and modestly elevated in 6J after DOX treatment, reflecting the stronger inflammatory response in 6N.

In contrast, the 6J mice showed significant upregulation of *Serpina3n* (Fig. 7e) and *Lcn2* (Fig. 7f), acute-phase proteins commonly elevated during cellular stress responses [32], whereas their expression remained unchanged in 6N mice. *Chil3*, a gene linked to immune modulation and tissue remodeling, was upregulated in both substrains following DOX treatment, with substantially higher expression in 6J mice (Fig. 7g). Finally, *Retnla*, a gene associated with tissue repair and M2 macrophage-mediated remodeling, was significantly downregulated in both 6N and 6J (Fig. 7h), indicating a shared suppression of pro-reparative and metabolic processes following DOX exposure. Collectively, these gene expression patterns reinforce the distinct, substrain-specific molecular strategies engaged in response to DOX treatment, with 6N displaying altered inflammatory and cellular stress responses, while 6J primarily activates metabolic and oxidative stress defense mechanisms to mitigate cardiotoxicity.

### Substrain-Specific Effects of DOX on Myocardial Fibrosis in C57BL/6 Mice

**Fig. 8:**
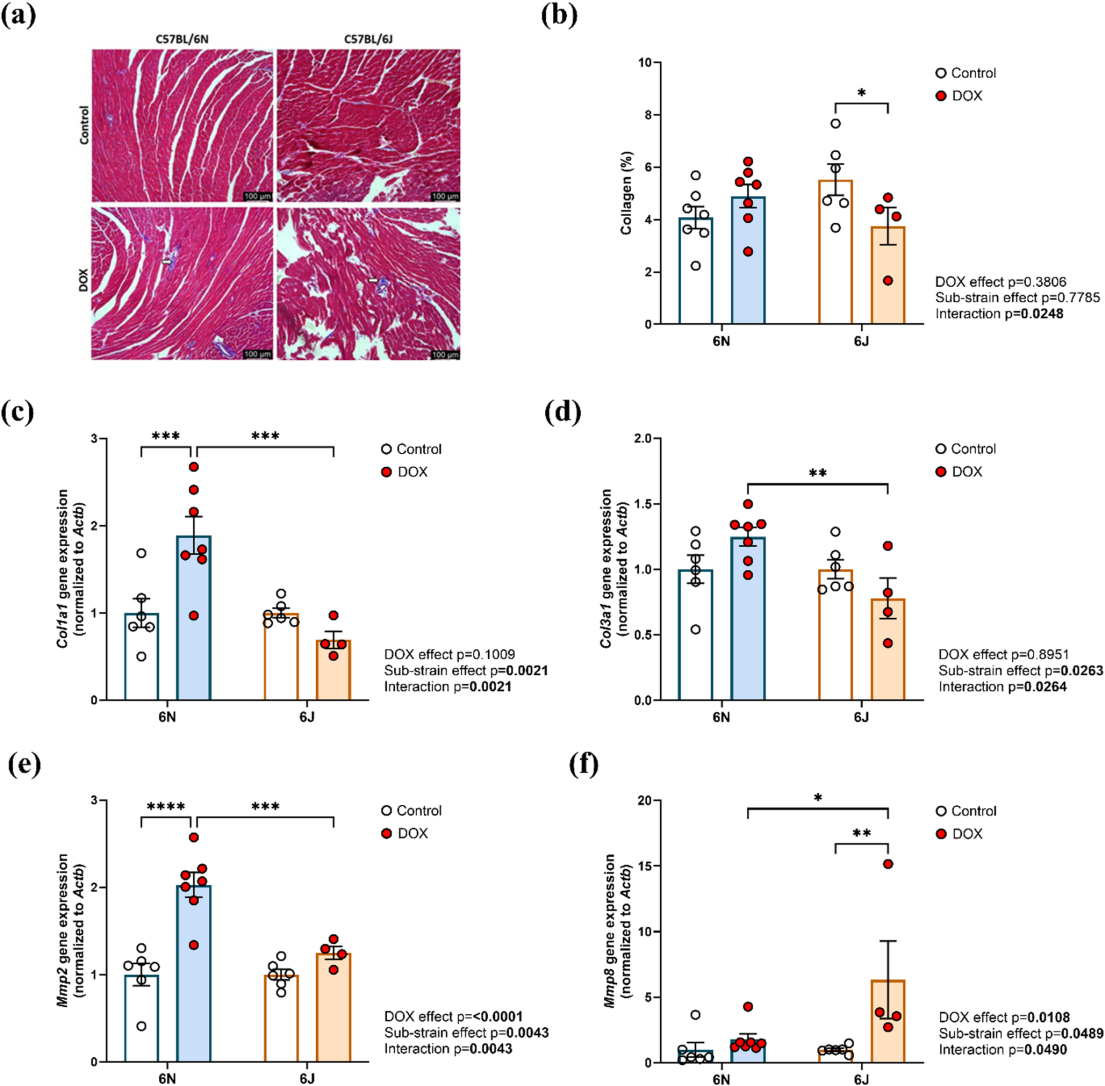
Substrain-specific differences in DOX-induced extracellular matrix remodeling and collagen regulation in C57BL/6 mice. Representative images from **a** Masson’s trichrome stained heart ventricular sections, bar scale = 100 μm. Fibrotic areas are indicated with arrows. **b** Collagen content (%). The mRNA expression of **c** Collagen 1a1 (*Col1a1*), **d** Collagen 3a1 (*Col3a1*), **e** Matrix metalloproteinase-2 (*Mmp2*), and **f** Matrix metalloproteinase-8 (*Mmp8*), normalized to *Actb* (*β-actin*). Values are represented as means ± SEMs (*n* = 4–7 per group). Statistical significance of pairwise comparisons was determined by two-way ANOVA with Tukey’s post-hoc analysis as appropriate (*p < 0.05, **p < 0.01, ***p < 0.001, ****p < 0.0001). Detailed statistical analyses are provided in Supplementary Table 3

Histopathological analysis using Masson’s trichrome staining and quantitative assessment of collagen content revealed a significant reduction in collagen deposition in DOX-treated 6J mice, whereas no significant change was observed in 6N mice compared with their respective controls (Fig. 8a, b). These histopathological differences were also reflected at the transcriptional level. Expression of Collagen type I alpha 1 (*Col1a1*) was significantly increased in 6N mice treated with DOX, while it was reduced in 6J mice compared to their controls (Fig. 8c). Similarly, Collagen type III alpha 1 (*Col3a1*) expression was elevated in 6N mice following DOX treatment, whereas it was lower in 6J mice (Fig. 8d). Matrix metalloproteinase-2 (*Mmp2*) expression was significantly upregulated in 6N mice and slightly increased in 6J mice after DOX exposure (Fig. 8e). In contrast, Matrix metalloproteinase-8 (*Mmp8*) showed no difference in 6N mice, but was significantly upregulated in 6J mice following DOX treatment (Fig. 8f). Together, these results indicate that DOX treatment differentially modulates extracellular matrix remodeling signaling pathways between the two substrains.

## Discussion

The 6N and 6J are closely related mouse substrains but differ genetically and phenotypically [8, 9]. A key distinction is a spontaneous in-frame deletion in the *Nnt* gene in 6J, which disrupts mitochondrial NADPH production and redox balance, contributing to metabolic defects such as insulin resistance and obesity [10, 11]. In contrast, 6N carries null mutations in *Mylk3* and *Crb1 (rd8)*, both absent in 6J [12, 13]. These genetic differences, including over 50 coding and structural variants [6] can influence cardiovascular phenotypes, with 6N mice displaying age-related dilated cardiomyopathy linked primarily to *Mylk3* deficiency [12].

Our study provides a comprehensive evaluation of substrain-specific vulnerabilities to DIC in C57BL/6 mice, revealing significant differences between the 6N and 6J substrains. We observed that under saline treatment, 6N mice exhibited higher body weights than 6J, consistent with existing literature indicating increased adiposity and overall body mass in 6N mice under standard diet conditions, likely driven by distinct metabolic profiles and genetic factors beyond the *Nnt* mutation [33, 34]. The echocardiographic data indicates that only the 6J mice developed overt systolic dysfunction after DOX, with significant declines in EF and FS. Previous studies using comparable cumulative DOX doses (~ 24 mg/kg) have also reported marked declines in EF and FS. However, those investigations were conducted exclusively in the 6J substrain ​ [27]. To our knowledge, this is the first study to directly compare both 6N and 6J substrains, revealing substrain-specific differences in susceptibility to DOX-induced systolic dysfunction. Importantly, both substrains exhibited a significant reduction in CO and SV, likely resulting from DOX-induced cardiac atrophy, as evidenced by the concomitant decreases in LV mass and HW/TL ratios, in line with reports that DOX triggers cardiac atrophy in mice​ [35]. However, there was no significant change in cardiomyocyte surface area in either substrain, suggesting that the observed atrophy may not result from reduced cell size but rather from loss of apoptotic cardiomyocytes or degradation of extracellular matrix components, as apoptosis-driven cardiomyocyte loss is a recognized mechanism contributing to DOX-induced cardiac atrophy and dysfunction [36]. Supporting this, gene expression analysis revealed a significant upregulation of the pro-apoptotic *Bax* gene in both 6N and 6J substrains, linking structural atrophy to activation of apoptotic pathways.

Natriuretic peptides such as *Nppa* and *Nppb* are well-established molecular markers of cardiac stress and injury, with elevated expression reported under various pathological conditions, including pressure overload, hypertrophy, and heart failure [37]. In our study, both 6N and 6J substrains showed significant *Nppa* upregulation after DOX treatment, consistent with its role as a key marker of maladaptive remodeling and oxidative stress in anthracycline-induced cardiomyopathy [2, 38]. The observed upregulation of *Nppb* in both sub-strains, reaching statistical significance only in 6N, further supports its role as a sensitive biomarker of DOX-induced cardiac injury linked to mitochondrial dysfunction and ROS accumulation [39]. In addition to natriuretic peptides, both substrains showed increased expression of *Myh6* and *Myh7*, representing an early compensatory phase remodelling, where the myocardium boosts the production of key contractile proteins to maintain function after DOX-induced damage. Instead of immediately switching from one myosin isoform to another, oxidative and mitochondrial stress caused by DOX can temporarily increase both *Myh6* and *Myh7*, which are adjacent in the genome and share common regulatory control [40, 41]. Additionally, heterogeneous responses among cardiomyocyte subsets may yield a mixed expression pattern, with both genes upregulated at the tissue level [42]. Despite genetic differences, both substrains showed similar expression levels of *Nppa*,* Nppb*,* Myh6*, and *Myh7* indicating no substrain-specific transcriptional response for these genes.

GSEA provided deeper insights into the molecular mechanisms underlying these observations. In the 6N substrain, DOX treatment resulted in significant enrichment of pathways such as TNFα signaling via NF-κB, TGF-β signaling, and apoptosis, collectively indicating pathological remodeling process involving inflammation, fibrosis, and cardiomyocyte stress or injury. TNFα is a pro-inflammatory cytokine that, through activation of NF-κB, promotes the expression of inflammatory genes in cardiomyocytes [43]. NF-κB, a key transcription factor that regulates inflammatory processes, has been shown to be activated in the failing human heart with enhanced expression of proinflammatory cytokines [44]. Supporting these pathway-level findings, *S100a8* and *S100a9* were significantly upregulated in 6N mice following DOX treatment, indicating a stronger inflammatory response. These alarmins promote leukocyte recruitment and activate TLR-4/RAGE–NF-κB signaling, amplifying cytokine release and contributing to heightened inflammation in 6N mice [45, 46]. The inflammatory predisposition of 6N mice is further emphasized by the differential expression of *Ptgs2* (*COX-2*), a key mediator of cardiac stress and inflammation [47]. Together, the enrichment of inflammatory pathways and upregulation of genes such as *S100a8/a9* and *Ptgs2* illustrate a heightened inflammatory response in 6N mice following DOX exposure. In contrast, IL-6–JAK–STAT3 signaling was suppressed in 6N mice following DOX treatment, likely due to cross-regulation with NF-κB and STAT3 signaling. STAT3, a pleiotropic transcription factor activated by IL-6, plays essential cardioprotective roles by promoting anti-apoptotic, antioxidant, and metabolic functions [48]. DOX treatment has been shown to deactivate STAT3 while activating the pro-inflammatory STAT1, which is associated with interferon signaling [49]. The loss of STAT3 activity can lift restraints on inflammation, permitting NF-κB-driven responses to proceed unchecked [48]. Thus, DOX may create a pathological imbalance: heightened NF-κB/TNF-α signaling alongside impaired IL-6/STAT3 signaling, driven by mutual antagonism and the loss of STAT3’s broader cardioprotective roles.

The enrichment of TGF-β signaling pathway following DOX treatment in 6N substrain suggests activation of canonical profibrotic and remodeling mechanisms. TGF-β, a key regulator of cardiac fibrosis, promotes fibroblast-to-myofibroblast transformation via Smad2/3 activation, leading to increased synthesis of extracellular matrix (ECM) components such as collagen and fibronectin and suppression of ECM degradation [50]. Even when histopathology shows no obvious fibrosis after DOX treatment, the upregulation of fibrosis-associated genes such as *Col1a1* and *Col3a1* following DOX treatment indicates early transcriptional activation of ECM remodeling. This aligns with prior studies showing that DOX triggers a sequential response, initial rise in inflammatory and profibrotic mediators, followed by induction of collagen genes days later, before structural changes are histologically detectable [51]. Elevated *Mmp2* expression further supports this early remodeling phase, as *Mmp-2* is rapidly upregulated after DOX and contributes to matrix degradation during a period of heightened ECM turnover [52]. This dynamic remodeling state precedes chronic fibrosis, where MMP activity declines and collagen accumulates [52]. In contrast, 6J mice did not show activation of these profibrotic pathways. Instead, they exhibited reduced expression of collagen genes accompanied by increased *Mmp8* levels, indicating greater matrix degradation [53]. This distinct response is interesting, as it suggests that enhanced MMP activity in 6J mice may limit collagen accumulation, revealing a potentially different remodeling mechanism following DOX-induced injury. The significant downregulation of *Retnla* in both substrains post-DOX exposure indicates a shared response to anthyracycline-induced stress. *Retnla* (Resistin-like alpha) is a member of the resistin family and known to modulate fibrosis and inflammation [54]. Recent transcriptomic studies in mouse models of DOX cardiotoxicity have identified *Retnla* as significantly downregulated in the heart following DOX exposure. A 2024 analysis reported a 2-fold reduction in *Retnla* expression after 3 weeks of chronic DOX treatment [55]. This downregulation coincided with reduced expression of other M2 macrophage markers, indicating suppressed alternative macrophage signaling in DOX-induced cardiomyopathy [56]. Despite this similar transcriptional change, downstream remodeling responses diverged between substrains: 6N mice exhibited early upregulation of *Col1a1*,* Col3a1*, and *Mmp2*, suggesting an active profibrotic response, whereas 6J mice showed reduced collagen gene expression with elevated *Mmp8*, indicative of enhanced matrix degradation and impaired repair [53]. Long-term studies are warranted to determine whether these early divergent responses translate into distinct chronic outcomes such as fibrosis progression or ventricular dilation.

In addition to ECM remodeling, activation of apoptotic pathways was evident in both 6N and 6J substrains, marked by increased expression of the pro-apoptotic gene *Bax*. This molecular profile aligns with the cardiac atrophy, suggesting that increased apoptotic activity may underlie the observed decline in myocardial mass by promoting cardiomyocyte loss. Furthermore, the pronounced upregulation of *Sprr1a* in 6N hearts, compared to in 6J suggests a substrain-specific molecular response to DOX. *Sprr1a*, a pro-apoptotic/profibrotic gene, has been implicated in exacerbating fibrosis, apoptosis, and cardiac dysfunction in various pathological contexts. Previous studies have shown that *Sprr1a* overexpression promotes cardiac fibrosis/apoptosis while its knockdown alleviates these effects, particularly after myocardial infarction (MI) or ischemia/reperfusion injury [57, 58].

The 6J substrain also revealed enrichment of metabolic and oxidative stress pathways, including xenobiotic metabolism, heme metabolism, oxidative phosphorylation, and ROS signaling. Supporting this, qRT-PCR demonstrated significant upregulation of *Serpina3n* and *Lcn2* in DOX-treated 6J mice. These genes encode acute-phase proteins that are well-characterized markers of cellular stress and tissue injury, and their upregulation has been consistently reported across multiple models of organ damage and fibrotic pathologies [32]. The marked upregulation of *Lcn2* in 6J mice underscores its key role in regulating iron homeostasis and mitigating oxidative stress during DOX exposure. By sequestering excess iron and stabilizing redox balance, *Lcn2* helps reduce oxidative damage and apoptosis. Its induction aligns with the metabolic reprogramming observed in 6J, suggesting a protective role against iron-driven cardiotoxicity [59]. In line with this oxidative stress response, both substrains showed upregulation of *Chil3* (*Chitinase-3-like protein 1*), with a stronger increase in 6J. As a hypoxia-responsive gene, *Chil3* supports antioxidant defense and immune regulation [60]. The enhanced upregulation in 6J aligns with the observed enrichment of hypoxia-responsive pathways and oxidative stress pathways in this substrain, suggesting a more adaptive response to DOX-induced stress. Despite these adaptive molecular responses, the Hedgehog signaling pathway was downregulated in both 6N and 6J mice treated with DOX. Hedgehog signaling pathway regulates cardiac development angiogenesis, and repair after injury. Its downregulation with DOX indicates impaired angiogenic response and regenerative capacity [61]. There is also downregulation of the myogenesis pathway in 6N and the angiogenesis pathway in 6J, further highlighting strain-specific differences in cardiac repair mechanisms.

The study has a few limitations. First, mortality may introduce survival bias in the molecular and histological analyses, a common limitation in preclinical cardiotoxicity and heart failure models [62, 63]. Second, time-course analyses to evaluate fibrosis progression and gene expression dynamics would have provided deeper mechanistic insights, but this was not feasible within the scope of the current study. Third, the mechanisms underlying the substrain differences in metabolic responses to DOX are beyond the scope of this project. Future studies should investigate metabolic and hormonal regulators such as leptin, ghrelin, and PPAR signaling to better understand the mechanisms underlying the differential body composition changes and their potential impact on cardiac outcomes. Additional studies will focus on elucidating the causal role of *Nnt* and *Mylk3* mutations in driving the distinct phenotypes observed between 6N and 6J substrains. Together, these studies will help establish mechanistic links between genetic, metabolic, and physiological differences in 6N and 6J mice.

In conclusion, our study reveals distinct substrain-specific responses to DIC in 6N and 6J mice. These findings suggest that 6N mice are more prone to maladaptive inflammatory, fibrotic, and apoptotic remodeling, whereas 6J mice are more susceptible to oxidative injury and apoptosis-driven contractile failure. Collectively, our results highlight the critical role of substrain selection in preclinical models of anthracycline cardiotoxicity and support the use of genetically informed strategies when evaluating cardioprotective interventions.

## Supplementary Information

Below is the link to the electronic supplementary material.

Supplementary Material 1

Supplementary Material 2

## Data Availability

Data will be made available upon reasonable request.
