# Supplementary material for: Substrain-Dependent Differences in Doxorubicin-Induced Cardiotoxicity in Adult C57BL/6 Mice
Source: Cardiovasc Toxicol. 2025 Dec 23;26(1):4. doi: 10.1007/s12012-025-10076-6 (PMC12727845; doi:10.1007/s12012-025-10076-6)
Supplement: Supplementary file 1 — Supplementary Material 1 [file 12012_2025_10076_MOESM1_ESM.docx]

**Supplementary material-1**

**Table 1. Primer sequences used in this study.**

| **Gene** | **Forward Primer (5’ – 3’)** | **Reverse Primer (5’ – 3’)** |
| --- | --- | --- |
| ***Nppa*** | GGAGCCTACGAAGATCCAGC | TCCAATCCTGTCAATCCTACCC |
| ***Nppb*** | AGTCCTTCGGTCTCAAGGCA | CCGATCCGGTCTATCTTGTGC |
| ***Myh6*** | CTGCTGGAGAGGTTATTCCTCG | GGAAGAGTGAGCGGGGCATCAAGG |
| ***Myh7*** | TGCAAGGCTCCAGGTCTGAGGGC | GCCAACACCAACCTGTCCAAGTTC |
| ***S100a8*** | TGCCCTCTACAAGAATGACT | AAGCTCTGCTACTCCTTGTG |
| ***S100a9*** | CGACACCTTCCATCAATACT | TCAGCATCATACACTCCTCA |
| ***Sprr1a*** | CAAGGCACCTGAGCCCTGCAA | AGGCTCTGGTGCCTTAGGTTGG |
| ***Ptgs2/COX-2*** | CTGGTGCCTGGTCTGATGATG | GGCAATGCGGTTCTGATACTG |
| ***Serpina3n*** | GGGATGATCAAGGAACTGGTCT | CCGCGTAGAACTCAGACTTGAA |
| ***Lcn2*** | CCAGTTCGCCATGGTATTTT | TCCTTCAGTTCAGGGGACAG |
| ***Chil3*** | CAGGTCTGGCAATTCTTCTGAA | GTCTTGCTCATGTGTGTAAGTGA |
| ***Retnla*** | CCAATCCAGCTAACTATCCCTCC | ACCCAGTAGCAGTCATCCCA |
| ***Actb*** | TATTGGCAACGAGCGGTTCC | GGCATAGAGGTCTTTACGGATGTC |

**Table 2: Cardiac function measured by trans-thoracic echocardiography in Control and DOX treated mice**

| Parameter | Control /6N (n=7) | | N | DOX/6N (n=8) | | N | Control/6J (n=6) | | N | DOX/6J  (n=6) | | N | DOX effect | | Sub-strain effect | | Interaction effect | |
| --- | --- | --- | --- | --- | --- | --- | --- | --- | --- | --- | --- | --- | --- | --- | --- | --- | --- | --- |
|  | Mean | SEM |  | Mean | SEM |  | Mean | SEM |  | Mean | SEM |  | Effect size (%) | P value | Effect size (%) | P value | Effect size (%) | P value |
| Final body weight | 32.14 | 1.406 | 7 | 24.529 | 0.887 | 7 | 28.450 | 0.836 | 6 | 23.450 | 1.065 | 4 | 52.160 | <0.0001 | 7.463 | 0.0512 | 2.24 | 0.2692 |
| Final fat mass | 4.23 | 0.86 | 7 | 0.522 | 0.139 | 7 | 1.896 | 0.315 | 6 | 0.932 | 0.48 | 4 | 33.67 | 0.0006 | 5.725 | 0.108 | 11.63 | 0.0268 |
| Final fat free mass | 26.90 | 0.62 | 7 | 23.35 | 0.782 | 7 | 25.438 | 0.609 | 6 | 23.47 | 1.52 | 4 | 32.03 | 0.0037 | 1.913 | 0.4323 | 2.635 | 0.3581 |
| Ejection fraction (%) | 47.16 | 2.505 | 7 | 41.564 | 2.412 | 7 | 53.944 | 3.087 | 6 | 43.983 | 3.542 | 4 | 23.380 | 0.0137 | 8.169 | 0.1258 | 1.836 | 0.4577 |
| Fractional shortening (%) | 23.61 | 1.483 | 7 | 20.221 | 1.347 | 7 | 27.725 | 2.013 | 6 | 21.488 | 2.035 | 4 | 24.520 | 0.0114 | 7.686 | 0.1346 | 2.154 | 0.4189 |
| Cardiac output (ml/min) | 17.22 | 1.098 | 7 | 13.679 | 0.728 | 7 | 18.970 | 1.183 | 6 | 11.579 | 1.829 | 4 | 51.290 | 0.0001 | 0.05389 | 0.8808 | 6.342 | 0.115 |
| Stroke volume (ul) | 41.85 | 1.640 | 7 | 32.705 | 1.671 | 7 | 40.167 | 1.841 | 6 | 28.773 | 3.169 | 4 | 55.980 | <0.0001 | 4.188 | 0.174 | 0.668 | 0.5797 |
| Heart rate (beats/min) | 409.47 | 9.992 | 7 | 422.615 | 25.326 | 7 | 470.805 | 9.480 | 6 | 398.898 | 45.250 | 4 | 6.523 | 0.2092 | 2.674 | 0.4159 | 13.67 | 0.075 |
| LV mass (mg) | 116.85 | 2.913 | 7 | 99.445 | 2.944 | 7 | 121.835 | 6.731 | 6 | 101.683 | 5.492 | 4 | 43.090 | 0.0006 | 1.593 | 0.4446 | 0.2304 | 0.7698 |
| HW/TL(mg/mm) | 5.58 | 0.102 | 7 | 4.720 | 0.116 | 7 | 5.515 | 0.183 | 6 | 4.465 | 0.299 | 4 | 62.340 | <0.0001 | 1.737 | 0.344 | 0.6272 | 0.5668 |
| LVAW;s(mm) | 1.21 | 0.053 | 7 | 1.145 | 0.047 | 7 | 1.352 | 0.050 | 6 | 1.256 | 0.053 | 4 | 7.928 | 0.1443 | 19.72 | 0.0265 | 0.3005 | 0.7704 |
| LVAW;d(mm) | 0.91 | 0.027 | 7 | 0.887 | 0.039 | 7 | 1.037 | 0.035 | 6 | 0.963 | 0.045 | 4 | 5.882 | 0.1945 | 23.93 | 0.0135 | 1.429 | 0.5157 |
| LVPW;s(mm) | 0.99 | 0.045 | 7 | 0.888 | 0.039 | 7 | 1.088 | 0.044 | 6 | 1.019 | 0.053 | 4 | 11.390 | 0.0743 | 19.6 | 0.0226 | 0.4609 | 0.7088 |
| LVPW;d(mm) | 0.72 | 0.012 | 7 | 0.677 | 0.024 | 7 | 0.743 | 0.041 | 6 | 0.736 | 0.047 | 4 | 3.082 | 0.4086 | 7.31 | 0.2085 | 1.598 | 0.5503 |
| LVID;s(mm) | 3.42 | 0.110 | 7 | 3.374 | 0.127 | 7 | 3.048 | 0.098 | 6 | 3.026 | 0.133 | 4 | 0.294 | 0.7732 | 30.42 | 0.0075 | 0.04193 | 0.9132 |
| LVID;d(mm) | 4.47 | 0.076 | 7 | 4.226 | 0.109 | 7 | 4.244 | 0.031 | 6 | 3.966 | 0.134 | 4 | 23.440 | 0.0112 | 20.28 | 0.0171 | 0.1226 | 0.8418 |
| Vol;s(ul) | 48.68 | 3.691 | 7 | 47.186 | 4.074 | 7 | 36.687 | 2.800 | 6 | 36.083 | 3.982 | 4 | 0.253 | 0.7885 | 30.71 | 0.0072 | 0.04569 | 0.9092 |
| Vol;d(ul) | 91.06 | 3.539 | 7 | 80.188 | 4.840 | 7 | 80.558 | 1.387 | 6 | 68.930 | 5.584 | 4 | 22.310 | 0.013 | 20.87 | 0.0159 | 0.02499 | 0.9283 |

Values are presented as mean ± standard error of the mean (SEM) (N=6-8 per group). Statistical significance was determined by two-way ANOVA followed by Tukey’s post-hoc analysis. P<0.05 is considered statistically significant and written in bold and underlines. LV, left ventricle; HW/TL, Heart weight to tibia length ratio; LVAW; s, LV anterior wall during systole; LVAW;d, LV anterior wall during diastole; LVPW;s, LV posterior wall during systole; LVPW;d, LV posterior wall during diastole; LVID;s, LV internal diameter during systole; LVID;d, LV internal diameter during diastole; Vol;s, LV volume during systole; Vol;d, LV volume during diastole;

**Table 3: Gene expression by quantitative real-time PCR**

| Parameter | Control /6N (n=7) | | N | DOX/6N (n=8) | | N | Control/6J (n=6) | | N | DOX/6J (n=6) | | N | DOX effect | | Sub-strain effect | | Interaction effect | |
| --- | --- | --- | --- | --- | --- | --- | --- | --- | --- | --- | --- | --- | --- | --- | --- | --- | --- | --- |
|  | Mean | SEM |  | Mean | SEM |  | Mean | SEM |  | Mean | SEM |  | Effect size (%) | P value | Effect size (%) | P value | Effect size (%) | P value |
| *Nppa* | 1.012 | 0.083 | 7 | 1.938 | 0.171 | 7 | 0.999 | 0.089 | 6 | 2.309 | 0.068 | 3 | 77.4 | <0.0001 | 1.987 | 0.2023 | 2.27 | 0.1742 |
| *Nppb* | 0.737 | 0.071 | 6 | 1.515 | 0.156 | 6 | 1.000 | 0.235 | 6 | 1.361 | 0.325 | 4 | 29.17 | 0.0104 | 0.2645 | 0.7883 | 3.906 | 0.3088 |
| *Myh6* | 1.000 | 0.131 | 6 | 1.497 | 0.172 | 6 | 1.000 | 0.072 | 6 | 2.009 | 0.190 | 4 | 56.99 | <0.0001 | 6.593 | 0.0922 | 6.598 | 0.0921 |
| *Myh7* | 0.951 | 0.160 | 7 | 4.201 | 0.861 | 7 | 1.000 | 0.075 | 6 | 5.167 | 0.863 | 3 | 64.4 | <0.0001 | 1.204 | 0.4215 | 0.9843 | 0.4665 |
| *S100a8* | 0.576 | 0.132 | 6 | 13.655 | 3.001 | 7 | 1.000 | 0.254 | 6 | 2.927 | 0.584 | 4 | 27.21 | 0.0009 | 12.83 | 0.0146 | 15.03 | 0.009 |
| *S100a9* | 0.624 | 0.149 | 6 | 16.482 | 3.995 | 7 | 1.000 | 0.205 | 6 | 6.588 | 1.689 | 4 | 35.33 | 0.0006 | 6.958 | 0.0828 | 8.103 | 0.0628 |
| *Sprr1a* | 0.669 | 0.128 | 6 | 5.319 | 1.283 | 7 | 1.000 | 0.326 | 6 | 2.627 | 0.671 | 4 | 32.4 | 0.0018 | 4.58 | 0.1881 | 7.516 | 0.0964 |
| *Ptgs2/COX-2* | 1.000 | 0.312 | 6 | 1.857 | 0.226 | 6 | 1.000 | 0.088 | 6 | 1.669 | 0.168 | 4 | 36.71 | 0.0037 | 0.5627 | 0.6849 | 0.5637 | 0.6847 |
| *Serpina3n* | 1.000 | 0.138 | 7 | 1.980 | 0.295 | 7 | 1.000 | 0.161 | 6 | 9.623 | 6.565 | 4 | 17.38 | 0.0365 | 11.01 | 0.0896 | 11.01 | 0.0896 |
| *Lcn2* | 1.000 | 0.117 | 7 | 6.072 | 0.973 | 7 | 1.000 | 0.203 | 6 | 26.171 | 14.189 | 4 | 29.6 | 0.004 | 13.07 | 0.0428 | 13.07 | 0.0428 |
| *Chil3* | 0.633 | 0.108 | 6 | 8.019 | 1.083 | 6 | 1.000 | 0.159 | 6 | 17.401 | 2.824 | 4 | 75.58 | <0.0001 | 12.69 | 0.0006 | 10.85 | 0.0012 |
| *Retnla* | 0.876 | 0.062 | 6 | 0.286 | 0.064 | 7 | 1.000 | 0.173 | 6 | 0.321 | 0.052 | 4 | 61.35 | <0.0001 | 0.9583 | 0.4717 | 0.2991 | 0.6862 |

Values are presented as mean ± standard error of the mean (SEM) (N=3-7per group). Statistical significance was determined by two-way ANOVA followed by Tukey’s post-hoc analysis. P<0.05 is considered statistically significant and written in bold and underlines. Nppa (Natriuretic Peptide A), Nppb (Natriuretic Peptide B), Myh6 (Myosin Heavy Chain 6), Myh7 (Myosin Heavy Chain 7), S100a8 (S100 Calcium-Binding Protein A8), S100a9 (S100 Calcium-Binding Protein A9), Sprr1a (Small Proline-Rich Protein 1A), Ptgs2/COX-2 (Prostaglandin-Endoperoxide Synthase 2 / Cyclooxygenase-2), Serpina3n (Serine Peptidase Inhibitor, Clade A, Member 3N), Lcn2 (Lipocalin 2), Chil3 (Chitinase-Like Protein 3), Retnla (Resistin-Like Alpha)
